# Treatment of Varicose Veins

**Published:** 1875-09

**Authors:** 


*Treatment of Varicose Veins.*—M. Rigaud, professor in the Faculty of Nancy, reports 140 cases treated successfully in the following manner: The vein is exposed to the atmosphere, and isolated from the neighboring parts by means of a ribbon or piece of adhesive plaster, and in about seven days is completely dessicated and obliterated—the wound through the integument healing without difficulty.—*New Orleans Medical and Surgical Journal.*

